# The trialists’ perspectives on the participant retention in mental health randomised controlled trials

**DOI:** 10.1186/1745-6215-16-S2-P116

**Published:** 2015-11-16

**Authors:** Paulina Szymczynska, Stefan Priebe

**Affiliations:** 1 Queen Mary University of London, London, UK

## 

Understanding the factors affecting participant attrition in trials is essential for ensuring ethical and effective conduct, appropriate power of the study and successful completion of data collection.

The purpose of this research is to explore factors affecting the retention of patients with mental health problems in non-pharmacological randomised controlled trials (RCTs). This paper reports on a qualitative study with staff working on trials aiming to investigate the processes and strategies employed in mental health RCTs.

Trialists from universities and research centres in the UK were recruited to take part in this qualitative study. 30 semi-structured interviews were conducted to understand the experiences of staff working on trials, the processes involved in planning and running RCTs and specific issues relevant to clinical research in mental health. The data were analysed using a framework analysis approach.

This paper will focus on what retention strategies are used in non-pharmacological mental health trials, how the issue of attrition is dealt with, and what trialists perceive to affect participants’ decisions about staying involved or ceasing their involvement.

